# Insights from an Annual Advanced International Men’s Health course

**DOI:** 10.1038/s41443-024-00839-0

**Published:** 2024-02-16

**Authors:** Nakamura S. Hana, Muhammed A. M. Hammad, Ghoniem M. Gamal, Yafi A. Faysal

**Affiliations:** Department of Urology, University of California, Irvine, CA USA

**Keywords:** Therapeutics, Psychology

## Introduction and the main issue

Medical training provides students and residents with various skills, but often lacks comprehensive education on sexual healthcare. Less than half of US medical schools offer a formal curriculum in sexual medicine, leaving many medical trainees feeling unprepared to provide sexual healthcare [1]. Treating and understanding men’s sexual health problems is more challenging without proper education in reproductive health practices. The approaches to men’s reproductive health and residency expectations are significantly different between renowned health organizations like the American Urological Association (AUA) and the European Association of Urology (EAU). The two organizations agree on basic practices and trainings on sexual health medicine but have discrepancies when it comes to Andrology as a specialty, calling for expert opinions [2]. For example, in Europe, Andrology is a subset of the Urology program, but in the United States (US), Andrology is regarded as a study separate from Urology [2]. Additionally, clinical conditions during training vary between Europe and the US, with the former inviting a wider range of specialists to gain andrology certification and the latter dedicating post-residency training and fellowships to those who choose to specialize in male infertility [2]. Thus internationally, there is a disconnect on men’s sexual health studies, and a lack of standardized academic resources for andrology.

The University of California Irvine (UCI) is addressing this issue by hosting a forum with global experts to clarify sexual health guidelines for medical trainees. The forum offers international, standardized, and accessible sexual health education that is imperative to the medical field today to further develop sexual medicine didactics. UCI offered their sixth Annual Advanced International Men’s Health course, a free virtual 2-day course approved for the American Medical Association Physician’s Recognition Award (AMA PRA) Category 1 credit (a verification of a physician’s participation in continuing medical education activities). The course provided training for specialists in men’s health, urinary incontinence, and andrology, and will be offered again next year.

Link: https://www.urology.uci.edu/uci-advanced-international-mens-health-course.shtml.

## New perspectives and its strengths

Since the UCI International Men’s Health Course first began in 2018, the curriculum has undergone polishing and upgrading over the years. The course in 2018 had 16 physicians from 5 different countries outside of the US and was an in-person cadaver and didactic program. Because of the Covid-19 pandemic, Men’s Health course was converted to a virtual format, which allowed the expansion of the curriculum—reaching more people globally. In fact, in 2023, the course included lectures from 63 guest faculty from 52 institutions across 16 states and 13 countries with over 1200 international registrants. Currently, the 2-day program covers male factor infertility, hypogonadism, Peyronie’s disease, penile prosthesis, lower urinary tract symptoms (LUTS), incontinence, chronic orchialgia, pelvic floor dysfunction, and female sexual dysfunction, among others.

## The implications for the future

The current standards for urology training focus on “medication of competences and sills in various fields of action for outpatient urology instead of persisting on the minimum and reference numbers of surgical and diagnostic procedures” [3] that leaves many fields of actions unrealized, including andrology [3]. This is because many institutions do not have every and all subspecialties of urology—creating a necessity for these institutions to cater to new residency training programs for distinctive specialties, such as in the case of andrology [3].

UCI’s Annual Advanced International Men’s Health course offers a chance to learn about andrology and improve sexual medicine teaching in institutions, contributing to the overall standardization of men’s health. The organizing committee plans to hold the seventh annual iteration of the UCI advanced International Men’s Health course on January 27 and 28, 2024. The course will feature 75 world-renowned faculty from 67 institutions and 18 countries, and it aims to enable participants to build the foundations of andrology as a subspecialty.
